# Dynamic Alterations of Spontaneous Neural Activity in Parkinson's Disease: A Resting-State fMRI Study

**DOI:** 10.3389/fneur.2019.01052

**Published:** 2019-10-01

**Authors:** Chao Zhang, Binru Dou, Jiali Wang, Kai Xu, Haiyan Zhang, Muhammad Umair Sami, Chunfeng Hu, Yutao Rong, Qihua Xiao, Nan Chen, Kuncheng Li

**Affiliations:** ^1^Department of Radiology, Affiliated Hospital of Xuzhou Medical University, Xuzhou, China; ^2^Department of Radiology, Affiliated 2 Hospital of Xuzhou Medical University, Xuzhou, China; ^3^Department of Neurology, Affiliated Hospital of Xuzhou Medical University, Xuzhou, China; ^4^Department of Radiology, Xuanwu Hospital, Capital Medical University, Beijing, China

**Keywords:** Parkinson's disease, resting-state fMRI, dynamic brain activity, amplitude of low-frequency fluctuations, support vector machine

## Abstract

**Objective:** To investigate the dynamic amplitude of low-frequency fluctuations (dALFFs) in patients with Parkinson's disease (PD) and healthy controls (HCs) and further explore whether dALFF can be used to test the feasibility of differentiating PD from HCs.

**Methods:** Twenty-eight patients with PD and 28 demographically matched HCs underwent resting-state functional magnetic resonance imaging (rs-fMRI) scans and neuropsychological tests. A dynamic method was used to calculate the dALFFs of rs-fMRI data obtained from all subjects. The dALFF alterations were compared between the PD and HC groups, and the correlations between dALFF variability and disease duration/neuropsychological tests were further calculated. Then, the statistical differences in dALFF between both groups were selected as classification features to help distinguish patients with PD from HCs through a linear support vector machine (SVM) classifier. The classifier performance was assessed using a permutation test (repeated 5,000 times).

**Results:** Significantly increased dALFF was detected in the left precuneus in patients with PD compared to HCs, and dALFF variability in this region was positively correlated with disease duration. Our results show that 80.36% (*p* < 0.001) subjects were correctly classified based on the SVM classifier by using the leave-one-out cross-validation method.

**Conclusion:** Patients with PD exhibited abnormal dynamic brain activity in the left precuneus, and the dALFF variability could distinguish PD from HCs with high accuracy. Our results showed novel insights into the pathophysiological mechanisms of PD.

## Introduction

Parkinson's disease (PD) is a common neurodegenerative disorder characterized by progressive impairment of motor function and widespread non-motor symptoms, which affects patients' quality of life and is, hence, a significant social burden (1–3). At present, the pathophysiological mechanism of PD is not fully understood, and it is still a neuroimaging challenge to form a definitive diagnosis at the early stage of the disease (1). Magnetic resonance imaging (MRI) has made great contributions in the clinical evaluation of PD (4, 5). Conventional MRI has been used to exclude secondary parkinsonism caused by neoplasms, vascular parkinsonism, and multiple sclerosis among others. The common imaging features of primary PD include iron deposition and substantia nigra atrophy (6, 7). However, several new reports have revealed that the cerebral region is widely involved in patients with PD. Therefore, it is important to explore novel imaging features that could help effectively identify PD.

In recent years, advanced neuroimaging techniques have allowed us to noninvasively explore the nature of the human brain in an efficient manner (8–10). Resting-state functional MRI (rs-fMRI) is an established tool to investigate the intrinsic neuronal activity of the human brain by measuring the amplitude of spontaneous low-frequency fluctuations (ALFFs) of blood-oxygen-level dependent (BOLD) signals (11–13). ALFF has been widely used to investigate regional brain activity in neurological diseases. Abnormal ALFFs in PD have been detected in extensive brain regions and act as an important characteristic related to subtypes of motor symptom or comorbidities (14). When compared with healthy controls (HCs), patients with tremor-dominant PD exhibited increased ALFF in the right cerebellar posterior lobe, while those with PD with postural instability/gait difficulty exhibited decreased ALFF in the bilateral putamen and cerebellar posterior lobe (15). PD patients with depression had significantly lower ALFF in the prefrontal cortex and anterior cingulated cortex than PD patients without depression (16). PD patients with apathy showed lower ALFF in the left orbital middle frontal gyrus and bilateral superior frontal gyrus (17) than PD patients without apathy. Further, PD patients with visual hallucinations showed lower ALFF in both lingual gyrii/cunei and greater ALFF in the temporo-parietal regions, medial temporal gyrus, and cerebellum than those with non-visual hallucinations and HCs (18). By measuring the local spontaneous brain activities, these studies provided satisfactory evidence that widespread cerebral regions were involved in PD, which greatly contributed to the understanding the neurobiological foundations of such disorders. However, the results of these studies were limited in that their focus on abnormal brain activities in PD with different motor/non-motor symptoms was based solely on group-level analysis; thus far, to our best knowledge, no study has used these abnormal features to distinguish PD with specific symptoms at an individual level.

Previous studies on aberrant static ALFF in PD are inconsistent as they report both lower and higher local brain activity in various cerebral areas. However, a static state analysis of ALFF ignores the dynamic characteristics of brain activity during the whole scanning period. Evidences from rs-fMRI that employ a sliding window approach have effectively detected dynamic functional connectivity features with higher sensitivity than the static state method (12, 19, 20). Recent studies reported that dynamic rs-fMRI analysis strategy not only made good contributions to human-brain exploration but also played an important role in studying the pathogenesis of schizophrenia (21–23). Nevertheless, few reports have focused on time-varying local spontaneous neuronal activity in PD, as ALFF itself exists with substantial fluctuations either (24, 25). Furthermore, support vector machine (SVM) is one of the most widely used supervised machine-learning approaches that can enable individual-level classification and prediction with high accuracy (26, 27). Uddin et al. (28) applied SVM to distinguish patients with autism from normal individuals with a classifier accuracy of over 80%. Accordingly, SVM has been proposed as an effective tool for diagnostic application in the clinic.

To date, no studies have evaluated the combined effects of dynamic ALFF and supervised machine-learning approaches on PD. Based on previous findings that a static ALFF has greater heterogeneity in PD and the proven property of dynamic methods, we hypothesized that dynamic intrinsic local spontaneous neuronal activity will show greater variability in subjects with PD than HCs, and that it may be another powerful index of rs-fMRI in exploring the underlying mechanisms of PD. We aimed to identify the cerebral regions that displayed abnormal dynamic local neuronal activity based on the voxel level of the whole brain. Furthermore, we applied SVM to observe whether the aberrant dALFF could be used as a feature to distinguish PD from HCs. This study may improve our understanding of the potential pathophysiological mechanism of PD. Moreover, we hope that this research can contribute to the clinical diagnosis of PD at an individual level.

## Materials and Methods

### Subjects

This study was carried out in accordance with the tenets of the Helsinki Declaration and approved by the local ethics committee of Xuzhou Affiliated Hospital, Xuzhou Medical University. Written informed consent was obtained from all subjects before participation in the study.

The project used a convenience sample of 31 hospitalized patients with PD who met the UK Bank diagnostic criteria for PD (29). All patients underwent neuropsychological tests such as the Mini Mental Status Examination (MMSE) and the Montreal Cognitive Assessment (MoCA) and motor impairment evaluation including the Unified Parkinson's Disease Rating Scale (UPDRS) and the Hoehn and Yahr (H-Y) stage when patients were off medication. The included patients had no history of mental illness or other neurological diseases. The exclusion criteria of the participants were MRI-confirmed brain abnormalities such as trauma, stroke, tumor, and infection and contraindications to MRI such as claustrophobia and implanted metal devices. In addition, the subjects with a history of drug and/or alcohol abuse and syncope were also excluded. All patients underwent routine treatment, and none of them received any other relevant interventions. Thirty-two age- and sex-matched healthy volunteers were included as HCs. Three patients and four HCs were excluded because of head motion artifacts. Finally, 28 patients with PD (15 male and 13 female, 59.17 ± 9.72 years old) and 28 HCs (14 male and 14 female, 58.18 ± 6.46 years old) were included for analysis. There were no significant intergroup differences with respect to age and sex (Table 1). All patients underwent functional MRI scanning when they were off medication; all the HCs also underwent the same protocol for MRI scanning and neuropsychological tests. All subjects were right-handed.

**Table 1:** Demographics and clinical data.

**Variable**	**PD (*N* = 28)**	**HCs (*N* = 28)**	***P***
Sex (M/F)	15/13	14/14	0.789^#^
Age (years)	59.17 ± 9.72	58.18 ± 6.46	0.794^*^
Duration of PD (years)	8.46 ± 2.92	N/A	N/A
UPDRS-III	29.1 ± 8.70	N/A	N/A
H-Y	2.02 ± 0.71	N/A	N/A
MoCA	24.39 ± 2.52	25.86 ± 1.73	0.015^*^
MMSE	27.64 ± 1.25	27.71 ± 1.24	0.831^*^
CV values	0.18 ± 0.04	0.13 ± 0.12	<0.001^*^

PD, Parkinson's disease; HCs, healthy controls; M, male; F, female; UPDRS-III, unified Parkinson's disease rating scale; H-Y, Hoehn and Yahr disability scale; MoCA, Montreal Cognitive Assessment; MMSE, Mini-Mental Status Examination.

Data are presented as the range and mean ± SD.

# The p-value was obtained using a chi-square test.

* *The p-value was obtained by a general linear mode analysis*.

### MRI Data Acquisition

All participants were scanned in a 3.0 Tesla MRI scanner (GE Medical Systems, Signa HD, Waukesha, WI) with an eight-channel head coil. During the scan, comfortable foam pads were used to stabilize the head of each subject to minimize head motion, and all subjects wore earplugs to reduce the noise from the MRI machine. Then, an echo-planar imaging sequence was employed to acquire resting BOLD images. The parameters of the protocol are as follows: time of repetition, 2,000 ms; time of echo, 30 ms; field of view, 220 mm × 220 mm; slice thickness, 3 mm; slice gap, 1 mm; voxel size, 3.4 mm × 3.4 mm × 4.0 mm; number of slices, 36; flip angle, 90°; and total volume of each subject, 185.

### Preprocessing of rs-fMRI Data

The rs-fMRI data preprocessing were carried out using data Processing and Analysis for (Resting-State) Brain Imaging (DPABI) (http://www.rfmri.org/dpabi) (30). The first 10 time points of each subject were excluded to stabilize the status and allow participants to adapt to the scanning condition. Slice timing was carried out on the remaining 175 volumes to correct time differences. Realignment was performed to correct head motion by using a Friston-24 model for individual-level correction, and any subject with a head maximum displacement >2 mm, maximum rotation >2.0°, or mean framewise displacement (FD) >0.3 was excluded. In our study, mean FD was set as a covariate for further group-level statistics to minimize the potential influences of head motion. Several covariates such as the Friston 24 head-motion parameters, cerebrospinal fluid signal, and white matter signal were regressed. Then, the processed volumes were normalized to the standard Montreal Neurological Institute (MNI) echo planar imaging (EPI) template with a voxel size of 3 mm × 3 mm × 3 mm. Finally, functional volumes were smoothed with 6-mm full width at half maximum. We did not carry out global signal regression of our data given that there is still some controversy regarding removal of the global signal (31–33).

### Dynamic ALFF Analysis

The analysis of dynamic amplitude of low-frequency fluctuations (dALFF) was carried out using Temporal Dynamic Analysis (TDA) toolkits based on DPABI (34). Before dALFF calculation, functional volumes were bandpass filtered (0.01–0.08 Hz) to minimize the influences of low-frequency drifts and fluctuations of the signal. The sliding window is an important parameter to capture dynamic spontaneous neural activities, and the proper window length is critical for dynamic analysis. Too small a window length may not allow robust estimation of dynamic changes, and too long a window length may not be able to detect dynamic activity. Previous studies provided the range of the appropriate window length as 10–75 TR, step = 1 TR) (12, 35). To maximize the statistical power, a moderate sliding window length of 50 TR (step = 1 TR) was selected. The post-processed 175 volumes of each subject were segmented into 126 windows in all. The ALFF was calculated in each sliding window. The standard deviation (SD) of ALFF values of each voxel across 126 windows was further calculated to assess the variability of ALFF. We also calculated the static ALFF containing the whole sliding window.

### Statistical Analysis

Two-sample *t*-test was used to observe intergroup differences in age and MoCA/MMSE scores. Sex-based group difference was evaluated using the chi-square test. A general linear mode (GLM) with age, sex, and mean FD as covariates was used to compare the difference of dALFF/ALFF between the PD and HC groups. Multiple comparisons were corrected using Gaussian Random-Field (GRF) method (voxel level, *p* < 0.001; cluster level, *p* < 0.05).

Partial correlation analysis was calculated between dALFF variability and disease duration/MoCA/MMSE/UPDRS/H-Y with age and sex as covariates (*p* < 0.05). All statistical analyses were performed using SPSS version 16 (SPSS Inc., Chicago, IL, USA).

### Support Vector Machine Analysis

The intergroup dALFF difference was used as the classification feature in this study. We then trained the SVMs by providing labeled observations, for which the classification results were known. To overcome the limitations of our samples, the leave-one-out cross-validation (LOOCV) method was applied to estimate the generalization ability of our classifier. To verify the ability of the validation strategy, we also made a classification comparison by introducing 10-fold cross-validations. Then, the total accuracy, sensitivity, and specificity were obtained to assess classifier performance.

A permutation test was used to evaluate the statistical significance of this classification accuracy (36). The permutation test was repeated 5,000 times, and during each time, the classifier randomly reallocated labels of PD and HC to the training subjects and repeated the entire classification process. The *p*-value was obtained after the total permutation was accomplished.

## Results

### Demographics and Clinical Data

The details of age, sex, and MoCA/MMSE scores are listed in Table 1. The results showed no significant difference in age (*p* = 0.652), sex (*p* = 0.789), and MMSE (*p* = 0.831) between the PD and HC groups. However, the MoCA score of the PD group was significantly lower than that of the HCs (*p* < 0.05).

### Differences in ALFF/Dynamic ALFF and Correlational Analysis

**Figure 1:**
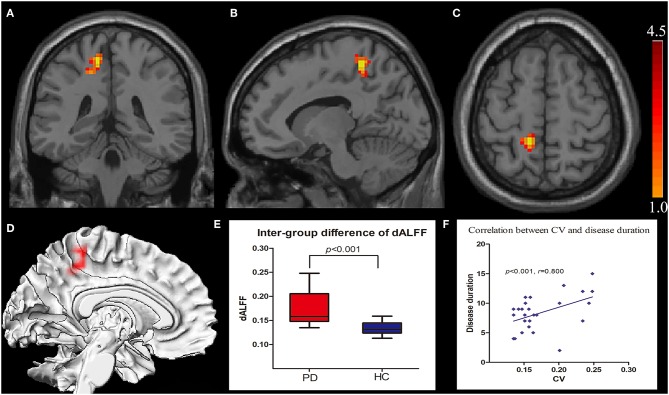
Increased CV of dALFF in the left precuneus displayed in coronal **(A)**, sagittal **(B)**, transverse **(C)**, and three-dimensional view **(D)**. Box plots with Whiskers (min–max) show the CV values in the left precuneus of the two groups **(E)**, and scatterplots show the relationship between the CV in the precuneus of the PD group and the disease duration **(F)**. CV, coefficient of variation; dALFF, dynamic spontaneous low-frequency fluctuation.

The intergroup differences in dALFF are shown in Figure 1 and Tables 1, 2. Compared with HCs, significantly increased coefficient of variation (CV) of dALFF was noted in the left precuneus of PD patients (*p* < 0.001). In addition, we found that the CV of dALFF was positively correlated with disease duration (*p* < 0.001, *r* = 0.800) (Figure 1), and no significant correlation was found between dALFF variability and MoCA/MMSE/UPDRS/H-Y scores (Supplementary Material). There were no significant intergroup differences in ALFF.

**Table 2:** dALFF alterations between PD groups and HCs.

**Region**	**Cluster size (voxel)**	**MNI (x,y,z)**	***t-*value**
Left Precuneus	94	(−12, −42, 60)	4.34

### Classification Results

**Figure 2:**
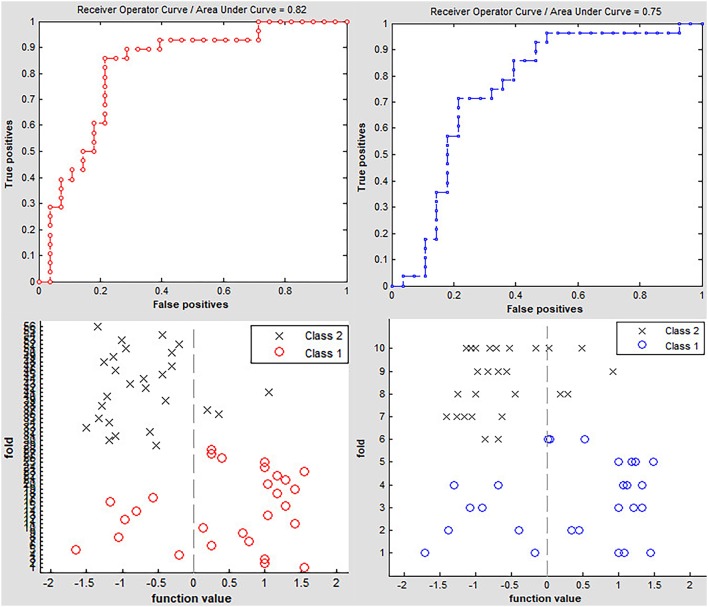
Classification accuracy of altered dynamic ALFF in the left precuneus obtained by the leave-one-out (red line) and nested 10-fold (blue line) cross validation methods in PD groups, respectively.

Classification results are shown in Figure 2. The accuracy of linear SVM classifier using LOOCV achieved an accuracy of 80.36%, sensitivity of 85.71%, and specificity of 75% (*p* < 0.001, non-parametric permutation approach). The receiver operating characteristic (ROC) curve of the classifier was 0.82. A 10-fold validation was also employed in our study to verify the reliability of the classification method, which generated a classifier accuracy of 71.43%.

## Discussion

Upon literature review, we observed that only few studies employed a TDA method to explore the neural-activity characteristics of PD. The present study showed the following findings: (1) the dALFF of patients with PD compared to HCs was notably different in the left precuneus; (2) a significant correlation between CV of dALFF in the left precuneus and the course of the disease was found in PD; (3) dALFF in the left precuneus showed high accuracy in distinguishing between patients with PD and HCs.

Prior studies have noted the importance of cerebral static local neural activities in PD (4, 14, 15). To our best knowledge, dynamic changes in spontaneous neural activity has been very poorly researched. The rs-fMRI analysis was based on the hypothesis that brain activity was in a stationary state during the entire scanning period; thus, the dynamic characteristics could not be identified. Dynamic algorithm was proven to represent the time-dependent characteristics of brain activity under the given scanning period. A recent study captured abnormal dALFF/ Regional Homogeneity (ReHo) in stroke patients by using TDA and compared it with HCs; they further found that variability in brain activity could be used to evaluate patients' motor function (24). Liu et al. (12) found abnormal functional network connection (FNC) through dynamic instead of static state. Dynamic FNC was significantly correlated with the frequency of epileptic seizures and the course of the disease. Furthermore, dynamic FNC could distinguish patients with idiopathic generalized epilepsy with generalized tonic–clonic seizures from controls with an accuracy of 77.91% through linear SVM classifier (*p* < 0.001). Dynamic algorithm showed the capability to characterize neural activity of the human brain by identifying specific function signatures.

Our dynamic algorithm showed that the dALFF in the left precuneus in PD was notably different than HCs. The result was partially consistent with previous reports that the left precuneus was a key cerebral region in patients with PD. Precuneus, which mainly constitutes the medial and posterior part of the parietal lobe, contributes to motor and cognitive tasks, and has been reported as displaying the highest resting metabolic rate among all cerebral regions (37). Interestingly, the precuneus consumed 35% more glucose than other brain regions in the resting brain (38). Perfusion single photon-emission computed tomography (SPECT) and [^18^F]fluorodeoxyglucose positron emission tomography ([^18^F]FDG-PET) proved that the precuneus was the most remarkable area of hypometabolism in the posterior cortical region (39). Another [^18^F]FDG-PET-based study on PD found that the metabolic capability of the left precuneus decreased with disease progression (40). Similarly, a more recent research using the arterial spin labeling (ASL) technique showed that cerebral blood flow (CBF) in the left precuneus significantly reduced in the PD group when compared with HCs (41). These functional imaging studies supported the view that the left precuneus might be more prone to attack in neuropsychiatric disorders. Additional rs-fMRI studies showed that the left precuneus was closely associated with motor and non-motor symptoms in PD. Hu et al. (42) found that increased local brain activity in the left precuneus was related to the Hamilton Depression Rating Scale score, by using static brain activity analysis. Thibes et al. (43) used a brain connection algorithm and showed that the left precuneus was a critical node connecting with specific cerebral regions in PD. In addition, morphological changes of the left precuneus were also found in PD with cognitive impairment and isolated apathy through voxel-based morphometry (44, 45). Therefore, the left precuneus is undoubtedly an important and vulnerable structure in patients with PD.

In our study, the higher variability of dynamic local brain activity level in the left precuneus was positively correlated with the course of PD. This meant that the degree of variation was significantly increased with an extended disease course, which reflected the increased or decreased brain activity at different sliding windows during the whole scanning period. These findings revealed a localized brain function impairment over time in PD. However, the abnormal dALFF did not correlate with UPDRS/H-Y scores in the present findings, likely because the heterogeneous motor symptoms in PD were associated with integration of multiple cerebral region function, rather than being determined by a single brain region impairment (46, 47). Unlike previous reports, the present study did not find significant intergroup differences with respect to ALFF, either because the sample size in our study was relatively small or because the result was not powerful enough to pass the multiple comparison correction of the present statistical methods. In fact, the dynamic features were concealed under the static analysis that represented a measure of the average amplitude of local activity across different scanning time points within the whole scan (12). Thus, static rs-fMRI may not be as sensitive as dynamic analysis to detect neural-activity changes. Our study indicated that dynamic analysis could completely unearth information of brain activity. In addition, the present result suggested that the left precuneus was an important structure involved in PD, and higher dALFF in this region was a promising imaging marker reflecting the disease duration. Besides, our findings did not show a correlation between dALFF and MoCA/MMSE tests. This may be because the MoCA and MMSE scales were mainly appropriate for cognitive screening, and our study lacked detailed assessment of cognitive performance compared to previous studies (48).

The imaging diagnosis of PD remains a challenge even now, as a confirmed diagnosis in most patients is still made depending on the clinical symptoms (49). An assessment of the iron content and volume of substantia nigra may be useful indicators to identify PD and evaluate the disease progression (6, 7). However, this approach has not been widely applied in the clinical management of patients with PD. Previous reports demonstrated that SVM was a powerful tool utilizing imaging features to distinguish PD patients from HCs. In our study, we tested the inter-group difference of dALFF in the left precuneus as a classification feature to discriminate PD from HCs through a linear SVM classifier. The accuracy of this classification was 80.36% when an LOOCV method was employed (non-parametric permutation correction, *p* < 0.001). Further, to compare the performance of SVM using LOOCV, a nested 10-fold cross-validation method was used to assess the classifier's performance; the accuracy was 71.43%. These findings showed that SVM could achieve better classification capability with LOOCV, and the results also provided evidence that patients with PD could be distinguished from HCs at the individual level when using dALFF variation in the left precuneus. These results support the hypothesis that the dALFF could identify individual PD patients.

Our study has some limitations. First, all patients were on medication. Although patients underwent fMRI scanning while they were off medication, the effects of the long-term treatment could not be completely ruled out. Second, the classification power based on the 28 PD patients was still not strong enough, and we just used SVM in the same sample to testify the classification accuracy. Third, the patients did not undergo comprehensive cognitive scales testing, which could have prevented a more accurate detection of cognitive performance. Future research should include a larger sample size and another independent test sample should be recruited for testifying classification accuracy.

## Conclusion

To our best knowledge, this is the first study to attempt to investigate the dynamic spontaneous neural activities in patients with PD. Our results provided evidence that dynamic analysis was more sensitive to detect alteration of brain activity than a static method. In addition, the CV of dALFF was found to be correlated with the course of the disease, which may ultimately contribute to identifying PD at the individual level. Thus, our results provide novel insights on the pathophysiological mechanisms of PD.

## Data Availability Statement

The datasets generated for this study are available on request to the corresponding author.

## Ethics Statement

The studies involving human participants were reviewed and approved by Ethical committee of Xuzhou Affiliated Hospital. The patients/participants provided their written informed consent to participate in this study.

## Author Contributions

CZ and BD contributed equally to this work for the conception/design of the study, the acquisition, analysis, interpretation of data, drafting of the manuscript, final approval of the version to be published, and agreement to be accountable for all aspects of the research. JW, CH, YR, HZ, and MS were responsible for data analysis, drafting of the manuscript, final approval of the version to be published, and agreement to be accountable for all aspects of the research. QX, NC, and KL were responsible for revision of the manuscript, final approval of the version to be published, and agreement to be accountable for all aspects of the work. KX was responsible for design of the study, revision of the work, final approval of the version to be published, and agreement to be accountable for all aspects of the work.

### Conflict of Interest

The authors declare that the research was conducted in the absence of any commercial or financial relationships that could be construed as a potential conflict of interest.
