# Supplementary material for: Dynamic Alterations of Spontaneous Neural Activity in Parkinson's Disease: A Resting-State fMRI Study
Source: Front Neurol. 2019 Oct 1;10:1052. doi: 10.3389/fneur.2019.01052 (PMC6779791; doi:10.3389/fneur.2019.01052)
Supplement: Supplementary file 1 [file Image_1.pdf]

Supplementary Material

1.1 Supplementary Figures

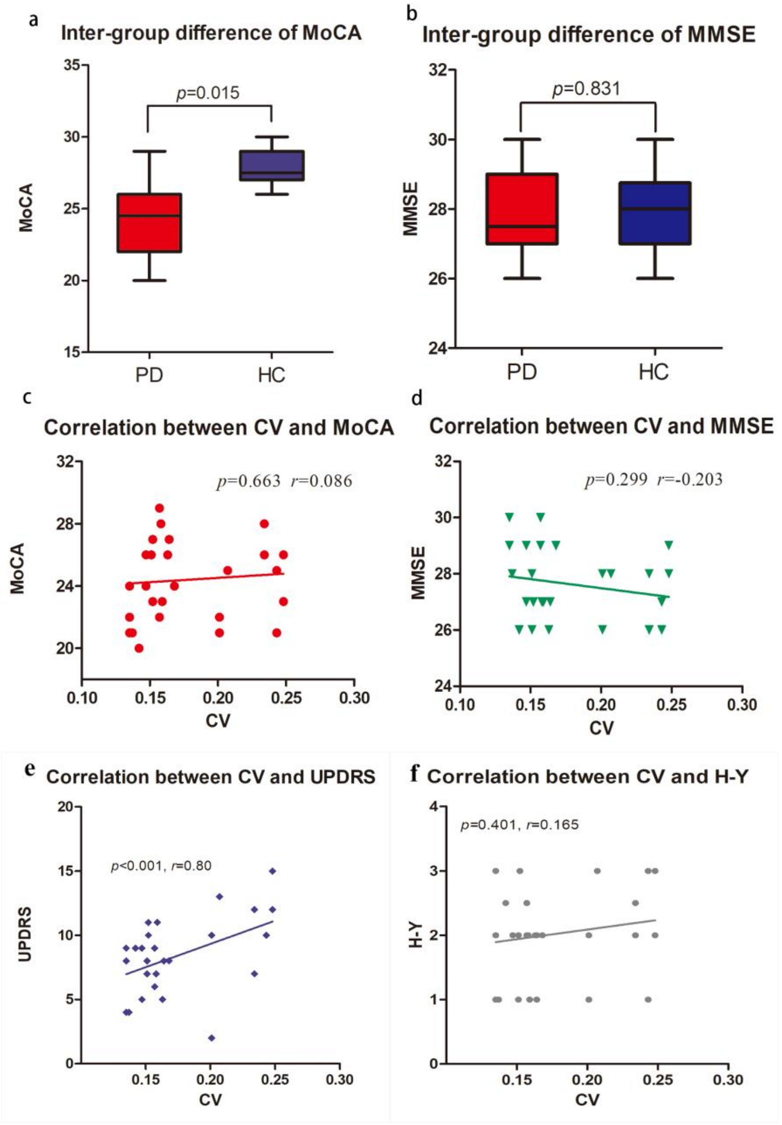

Supplementary Figure 1. Inter-group difference of MoCA(c)/MMSE(d)/UPDRS(e)/HY(f) scores between PD group (red) and healthy controls (blue). Box plots with Whiskers min to max show the MoCA/MMSE scores in the PD groups and healthy controls, respectively. Scatterplots show the relationship between the coefficient of variation of dALFF in the precuneus and MoCA/MMSE in PD groups.
